# Effects of bensulfuron-methyl residue on photosynthesis and chlorophyll fluorescence in leaves of cucumber seedlings

**DOI:** 10.1371/journal.pone.0215486

**Published:** 2019-04-23

**Authors:** Lanlan Sun, Hongle Xu, Hongdan Hao, Shiheng An, Chuantao Lu, Renhai Wu, Wangcang Su

**Affiliations:** 1 Institute of Plant Protection, Henan Academy of Agricultural Sciences, Henan Key Laboratory of Crop Pest Control, IPM key laboratory in the southern part of North China for Ministry of Agriculture, Zhengzhou, China; 2 College of Plant Protection, Henan Agricultural University, Zhengzhou, China; Hainan University, CHINA

## Abstract

A potted soil experiment was conducted to investigate the effects of bensulfuron-methyl (BSM) residue on the growth and photosynthesis of seedlings of a local cucumber variety (Xia Feng No.1). When the residue of bensulfuron-methyl in soil exceeded 50μg kg^-1^, it significantly inhibited the growth of cucumber, chlorophyll content and photosynthetic capacity of cucumber. BSM treatment caused significant decreases in the biomass, chlorophyll content, net photosynthesis rate, stomatal conductance, and transpiration rate, photosystem II (PSII) maximum quantum yield, actual quantum yield, photochemical quenching coefficient, and electron transport rate in cucumber seedlings, but increased the minimal fluorescence yield and dark respiration rate. Moreover, comparisons of the patterns of absorbed light energy partitioning revealed that the fractions of excess and thermally dissipated energy increased with rising concentrations of the BSM residue, but the fraction of PSII photochemistry declined. The BSM residues caused reversible destruction in the PSII reaction centers and decreased the proportion of available excitation energy used in PSII photochemistry. The results suggested that rice or wheat fields sprayed with BSM will not be suitable for planting cucumbers in succession or rotation.

## Introduction

Herbicides are necessary for weed control in intensive crop production, and play an important role in modernization of agriculture. Bensulfuron-methyl (BSM) has been used since the 1970s and now is widely used in paddy fields in the world. In China, BSM was first used in paddy fields to control sedge and broadleaf weeds, and now it is also used to control broadleaf weeds in wheat fields [1].

As a sulfonylurea herbicide, BSM is principally absorbed by roots and leaves, and can inhibit acetolactate synthase and the biosynthesis of branched-chain amino acids[2]. The herbicide shows high selectivity and has been used extensively for many years. However, its overutilization can have negative impacts on farming, such as damaging sensitive crops and creating herbicide-resistant weeds[1].

BSM degradation occurs mainly through chemical hydrolysis and microbial processes in soil. Levels of soil organic matter and the soil pH affect the degradation of BSM[1]. Thus, BSM may persist for a long period (over 100 d) when applied under specific climatic and/or soil conditions; it may be filtered into the environment and its residues may impair the rotation of other crops[3]. The safety interval of BSM is 90 d in the north and 80 d in the south of China. Herbicide persistence in soil influences the choice of herbicide because of rotational crop restrictions in the following growing season[2,4]. Chen reported that the BSM residue in the soil were 183.08 μg kg^-1^ after treatment 84d[5]. The soil residues of sulfonylureas have injured rotational crops such as soybean, peanut, maize, rape, tobacco, sunflower, sugar beet, and dry beans [6–10]. Different maize varieties have varying degrees of sensitivity to the BSM residue. BSM has a persistence of 150 d, and this high soil persistence combined with the sensitivity of certain crops have suggested that the risk of carryover on rotational crops is high[11].

In the field of herbicide injury assessment, photosynthetic characteristics and chlorophyll fluorescence are used to determine the degree of phytotoxicity of herbicides in plants, in addition to conventional methods such as morphological characteristics and yield measurements[12–13]. A previous study reported that imidazolinone may damage photosynthetic organs in rice before visible injury, lowering the crop’s photosynthetic capabilities[14]. Tomar *et al*. [15] found that the photosynthetic parameters of soybean significantly declined in the presence of anthracene. Su *et al*.[9–10] reported that photosynthetic parameters in soybean, peanut and maize were reduced to various degrees by bensulfuron-methyl residues in soil.

Among abiotic stresses, herbicide residue constitutes a major hazard to agriculture and is an important factor limiting crop rotation. The exposure of plants to herbicide residue induces many changes in morphological and physiological parameters. Herbicide residues are receiving increasing attention as they threaten crop yield and the safety of the environment, as well as human health[16]. A great deal of effort has been expended to solve these problems, such as detection of residues and biodegradation in soil[1–2,17]. Herbicide persistence can be a significant problem for producers due to injury to crops planted in succession or rotation[18]. We attempted to determine the photosynthetic characteristics and chlorophyll fluorescence parameters of cucumber seedlings affected by the BSM residue in soil.

To alleviate weed hazards and change the unitary structure of weed communities, farmers often use vegetables and rice in rotations. There have been few reports on the phytotoxicity of BSM in vegetables during rotations of rice or wheat with vegetables. The purpose of this study was to investigate the effects of the BSM residue on photosynthesis and growth in cucumber seedlings.

## Materials and methods

### Materials and BSM treatment

**Fig 1:**
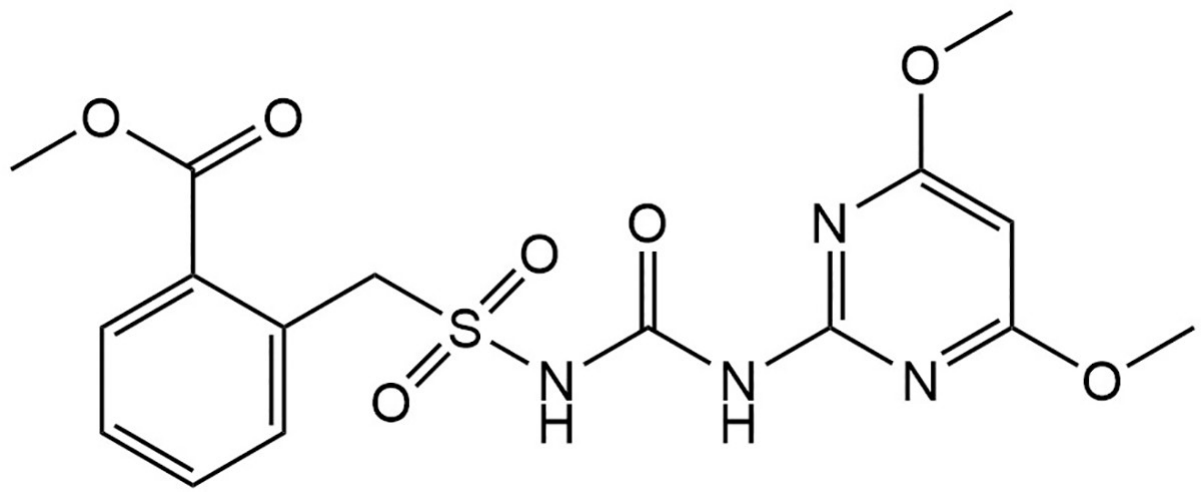
Chemical structure of bensulfuron-methyl.

Soil samples were collected from an uncultivated area at the Henan Academy of Agricultural Sciences in Xinxiang (35°18′N, 113°52′E), Henan Province, China, which have never been treated with herbicides. The soil samples were naturally air-dried and then passed through a 2 mm sieve. The soil samples was classified as Fluvo-aquic soil. The purity of BSM was 98.5% and the manufacturer was Hangzhou Tianyi Pesticide Manufactory, Zhejiang Province, China. Fig 1 shows the chemical structure of BSM.

The method of soil treatment was according to Su *et al*. [10]. Stock solutions of BSM were prepared by placing a known quantity of BSM in approximately 50 mL of acetone and then diluting with water to the 1 L mark in a volumetric flask. Standard solutions were created from the stock solutions to produce solutions with concentrations of 0.03125, 0.0625, 0.125, 0.25, 0.5 and 1.0 mg of active ingredient (a.i.) per liter of BSM. Two hundred grams of each soil sample was placed into pots (70 mm tall and 83 mm in diameter) and 40 mL of the standard solution was added to the untreated soil. For the control, 40 mL distilled water was added to the untreated soil. The soil samples were then manually mixed to ensure uniform distribution of the added BSM throughout the soil and allowed to equilibrate for 24 h. The BSM concentrations in the soil were 0, 6.25, 12.5, 25, 50, 100, and 200 μg active ingredient (a.i.) kg^-1^ soil. The recommended field application rate was 74 μg a.i. kg^–1^. The BSM concentration in the study refers to the concentration of the active ingredient.

Seeds of cucumber (*Cucumis sativus* L.) variety Xia Feng No.1 were obtained from Zhengzhou seed company. The seeds were pre-germinated for 24 h at 25°C. Five germinated seeds were sown in pots (70 mm tall and 83 mm in diameter) and 0.5 cm deep. The soil contained 5.5 g kg^–1^ organic matter, 29.8 mg kg^–1^ available nitrogen, 6.5 mg kg^–1^ available phosphorus, and 78.3 mg kg^–1^ available potassium. The soil pH was 8.4. The pots were watered regularly and the soil moisture was controlled to 20% by weighing. The pots were placed in a growth chamber under controlled conditions: 30/25°C day/night, 70%–75% relative humidity, and a 16 h photoperiod with a photosynthetic photon flux density (PPFD) of 150 μmol (photon) m^-2^ s^-1^[19]. The experiments were performed in a completely randomized design with four replications per concentration group in a greenhouse. After 14 d of BSM treatment, one cucumber seedling per pot was randomly selected to measure photosynthesis and chlorophyll fluorescence parameters. Each treatment was repeated four times. Growth parameters were determined on the basis of fifteen random samplings in four replications.

### Growth parameters

Shoot and root lengths were measured and recorded. The roots and shoots of each cucumber seedling were respectively put into paper bags, then fixed at 105°C for 30 min and dried at 80°C to a constant weight. The ratio of root to shoot (R:S) was calculated using the root dry mass (RDM) and shoot dry mass (SDM); total dry mass (TDM) was the sum of SDM and RDM.

### Relative chlorophyll content

A chlorophyll meter SPAD-502 (Konica Minolta Camera Co. Ltd., Japan) was used to measure the relative chlorophyll content. Each leaf was measured five times, and the average of five times of the same leaf was used as the leaf SPAD value. Each pot was measured in five plants, and there were four pots.

### Gas exchange and chlorophyll fluorescence

Photosynthetic rate and chlorophyll fluorescence were measured with a portable, open-flow gas exchange system (Li-6400; Li-Cor Inc., Lincoln, NE, USA) connected to a leaf chamber (Li-6400–40 leaf chamber fluorometer). The temperature, CO_2_ concentration, and PPFD in the leaf chamber were kept at 28 ± 0.5°C, 400 μmol mol^-1^, and 0 μmol (photon) m^-2^ s^-1^, respectively. Dark respiration rate (R_D_), minimal fluorescence (F_o_), and the maximum chlorophyll fluorescence yield (F_m_) were measured with weak modulated irradiation (<0.1 μmol m^-2^ s^-1^) using dark-adapted leaves. The maximum quantum yield of PS II photochemistry (F_v_/F_m_) was calculated as F_v_/F_m_ = (F_m_—F_o_)/F_m_.

We then commenced light activation. Net photosynthesis rate (*P*_N_), stomatal conductance (*g*_s_), intercellular CO_2_ concentration (*C*_i_), and transpiration rate (*E*) of single leaves were measured. The light intensity, temperature, and CO_2_ concentration in the leaf chamber were kept at 150 *μ*mol (photon) m^–2^ s^–1^, 28 ± 0.5°C, 400 *μ*mol mol^–1^, respectively. Simultaneously, the actual efficiency of PSII (Φ_PSII_), photochemical quenching coefficient (qP), and electron transport rate (ETR) were also measured. These parameters were calculated as follows: (1) Φ_PSII_ = (F_m_′—F_s_) /F_m_′; (2) qP = (F_m_′—F_s_)/(F_m_′—F_o_′); (3) ETR = Φ_PSII_ × PPFD × 0.5 × 0.84, where PPFD was the actinic light intensity, 0.5 was the proportion of light energy assigned to PSII, and 0.84 meant that 84% of the incident light was absorbed by leaves[20–21].

### Calculation of PSII efficiency and energy dissipation

The light energy absorbed by the photosynthetic system was dissipated into the fraction of photon energy absorbed by the PSII antennae (P), a thermally dissipated portion (D), and the remaining fraction (denoted as the “excess;” E), all estimated as percentages. The fraction of absorbed light energy dissipated thermally was calculated as D = 1—F_v_′/F_m_′ = 1 - (F_m_′—F_0_′)/F_m_′. The light energy utilized in the PSII photochemistry was estimated as P = ΔF/F_m_′ = (F_m_′—F)/F_m_′, and the excess was calculated from E = 1—P–D[22–23].

### Statistical analysis

Each data point was recorded four replicates repeatedly and was expressed as the mean ± standard deviation (SD). Mean values of photosynthesis and fluorescence parameters, plant growth, and plant biomass in shoots and roots were subjected to one-way analysis of variance (ANOVA) using SPSS 17.0 (SPSS, Chicago, IL, USA). Least significant difference (LSD) at p < 0.05 was used to compare the means.

## Results

### Shoot length, root length, and biomass changes in response to BSM residue stress

**Fig 2:**
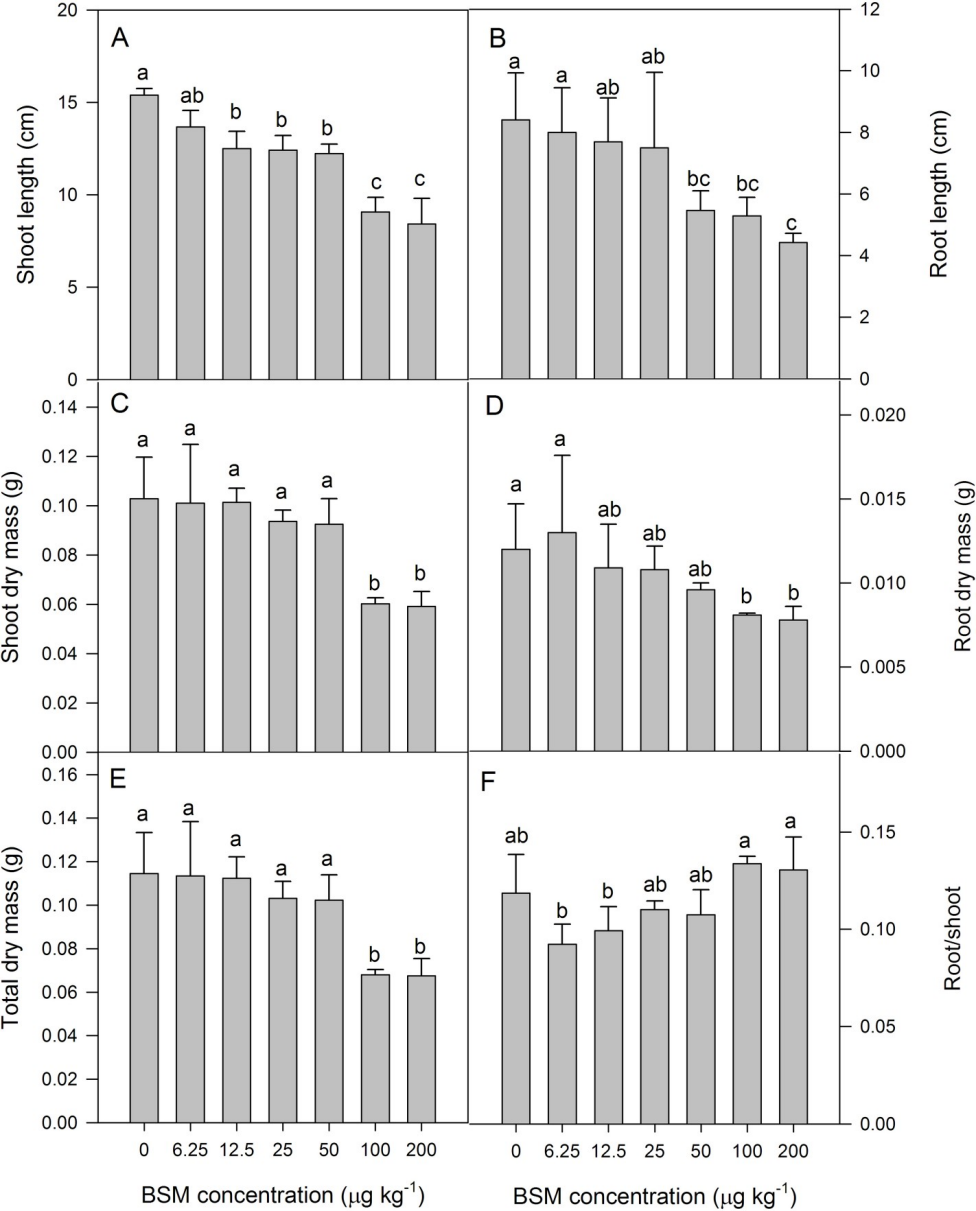
(A) Shoot length, (B) root length, (C) shoot dry mass, (D) root dry mass, (E) total dry mass, and (F) root: shoot ratio of cucumber seedlings under different concentrations (0, 6.25, 12.5, 25, 50, 100, and 200 μg kg^-1^) of the herbicide bensulfuron-methyl (BSM) in soil. Values are means of four replications, with error bars showing the SD. The means of each parameter were analyzed using Duncan’s multiple range test to check whether the differences between treatments were significant. The columns marked with different lower case letters indicate a significant difference between treatments at *p* < 0.05.

The growth of cucumber seedlings was greatly inhibited by the BSM residue. The effects of BSM residue on the biomass and the lengths of shoot and root of seedlings were shown in Fig 2. After treatment for 14 d, shoot length was not significantly different in the control vs. the BSM treatment of 6.25 μg kg^-1^. For BSM residue concentrations within the range of 12.5–200 μg kg^-1^, shoot length was significantly decreased by 18.84–45.35% (Fig 2A). Compared with the control, there were no significant differences in root lengths at BSM concentrations of 6.25, 12.5, and 25 μg kg^-1^. However, root length significantly decreased for concentrations of 50–200 μg kg^-1^, with inhibition rates of 35.18–47.43% (Fig 2B).

The SDM, RDM, and TDM of cucumber seedlings were decreased by BSM residue treatment (Fig 2C–2E). Compared with the control, the BSM residue concentrations of 6.25–50 μg kg^-1^ treatments did not have significantly different values of SDM, RDM and TDM; however, the 100 and 200 μg kg^-1^ treatments were significantly reduced by 33.61%,42.62%, respectively. The effect pattern of BSM on the R:S ratio varied; The R:S ratio of seedlings was decreased at BSM concentrations of 6.25 and 12.5 μg kg^-1^ but increased within the concentrations of 25–200 μg kg^-1^. Thus, BSM had a greater impact on RDM than SDM at lower concentrations. As the concentrations of the BSM residue increased, the growth of the above- and belowground parts of seedlings were severely suppressed.

### Gas exchange and chlorophyll fluorescence

**Fig 3:**
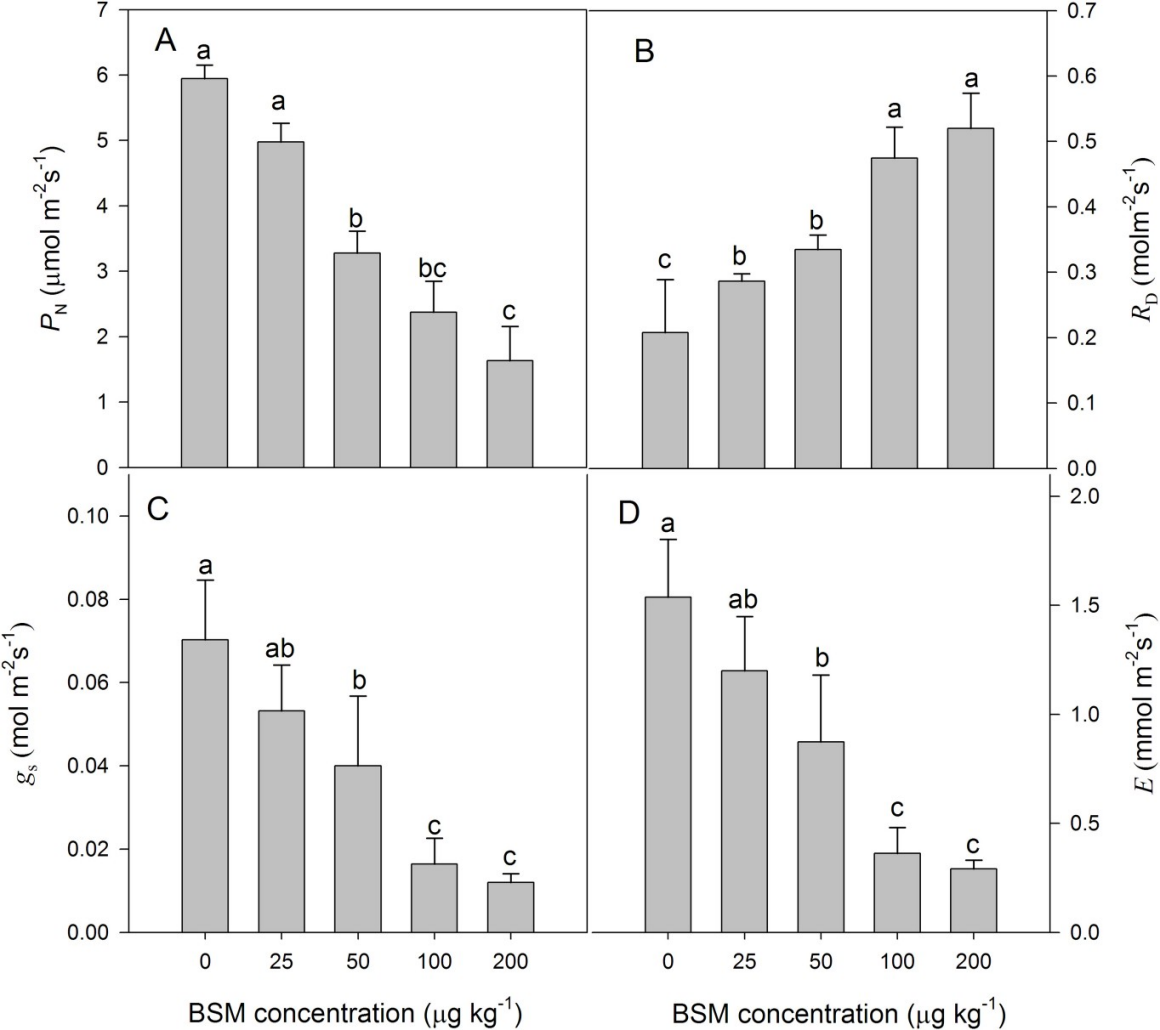
(A) Net photosynthetic rate, (B) respiration rate, (C) stomatal conductance, and (D) transpiration rate of cucumber seedlings under different concentrations (0, 25, 50, 100, and 200 μg kg^-1^) of the herbicide bensulfuron-methyl (BSM) in soil. Values are means of four replications, with error bars showing the SD. The means of each parameter were analyzed using Duncan’s multiple range test to check whether the differences between treatments were significant. Columns marked with different lower case letters indicate a significant difference between treatments at *p* < 0.05.

There were obvious decreasing trends in P_N_, g_s_, and E in the leaves during BSM treatment. Compared to the control, P_N_, g_s_, E, and R_D_ did not significantly differ at the BSM concentration of 25 μg kg^-1^. At concentrations of 50, 100, and 200 μg kg^-1^, the P_N_ values decreased by 45%, 60%, and 73%, respectively; and correspondingly the g_s_ values decreased by 42%, 77%, and 83%, and the E values decreased by 43%, 76%, and 81% (Fig 3A, 3C and 3D). The R_D_ values in the leaves rose gradually with an increase in BSM concentrations compared to the control. The *R*_D_ values at the concentrations of 50, 100, and 200 μg kg^-1^ increased significantly by 70%, 138%, and 168%, respectively (Fig 3B).

**Fig 4:**
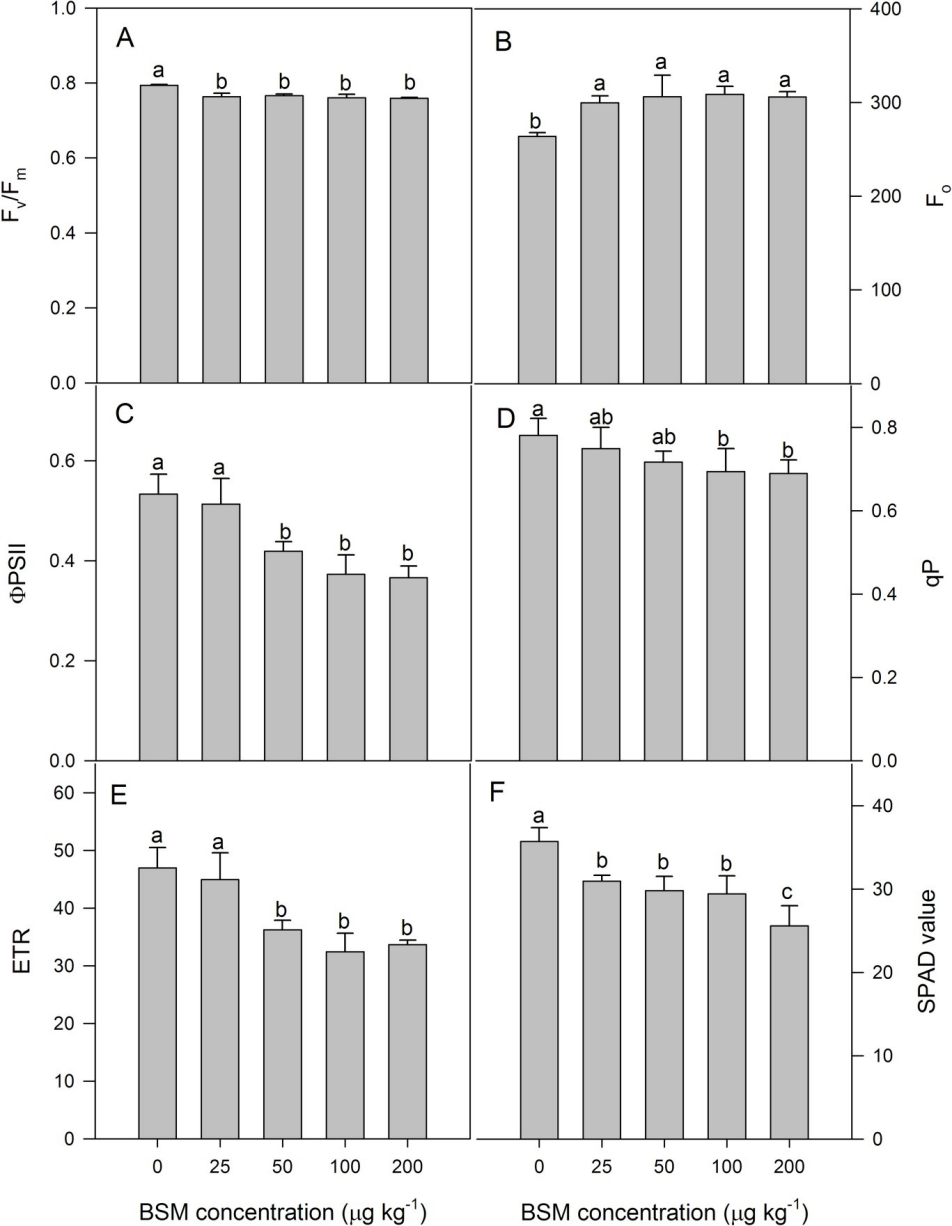
(A) Fv/Fm, (B) F_o_, (C) ΦPSII, (D) qP, (E) ETR, and (F) the SPAD value of cucumber seedlings under different concentrations (0, 25, 50, 100, and 200 μg kg^-1^) of the herbicide bensulfuron-methyl (BSM) in soil. Values are means of four replications, with error bars showing the SD. The means of each parameter were analyzed using Duncan’s multiple range test to check whether the differences between treatments were significant. Columns marked with different lower-case letters indicate a significant difference between treatments at *p* < 0.05.

The values of F_v_/F_m_ and F_o_ were affected by BSM residues and there was an obvious decreasing trend of F_v_/F_m_, but an increasing trend in F_o_, with increasing BSM residue concentrations (Fig 4A and 4B). For BSM treatment within the range of 25–200 μg kg^-1^, F_o_ increased by 14–17%.

Φ_PSII_ and ETR decreased as BSM residue concentrations increased (Fig 4C and 4E). Compared to the control, Φ_PSII_ and ETR of cucumber leaves at 50–100 μg kg^-1^ decreased by 21–31% and 23–31%, respectively (*p* < 0.05). At the concentration of 25 μg kg^-1^, there was no significant difference between the treatment and control in the Φ_PSII_ and ETR.

q_P_ provides an indication of the proportion of reaction centers that are open. The BSM treatment changed the q_P_ in cucumber seedlings after 14 d of treatment. Compared to the control, q_P_ of the leaves at 100 and 200 μg kg^-1^ decreased by 11% and 12%, respectively (*p* < 0.05). In contrast, the 25 μg kg^-1^ treatment showed no significant difference compared to the control (Fig 4D).

### Relative chlorophyll content

Different BSM concentrations treatments caused significant reductions in the SPAD values. At the BSM residue concentration of 6.25 μg kg^–1^, the SPAD value did not differ significantly from the control. As the BSM concentrations increased, the relative chlorophyll contents were greatly reduced. The inhibition of the relative chlorophyll contents at BSM concentrations of 12.5, 25, 50, 100, and 200 μg kg^-1^ in cucumber seedlings were 12%, 13%, 16%, 17%, and 28%, respectively (Fig 4E).

### PSII efficiency and energy dissipation

**Fig 5:**
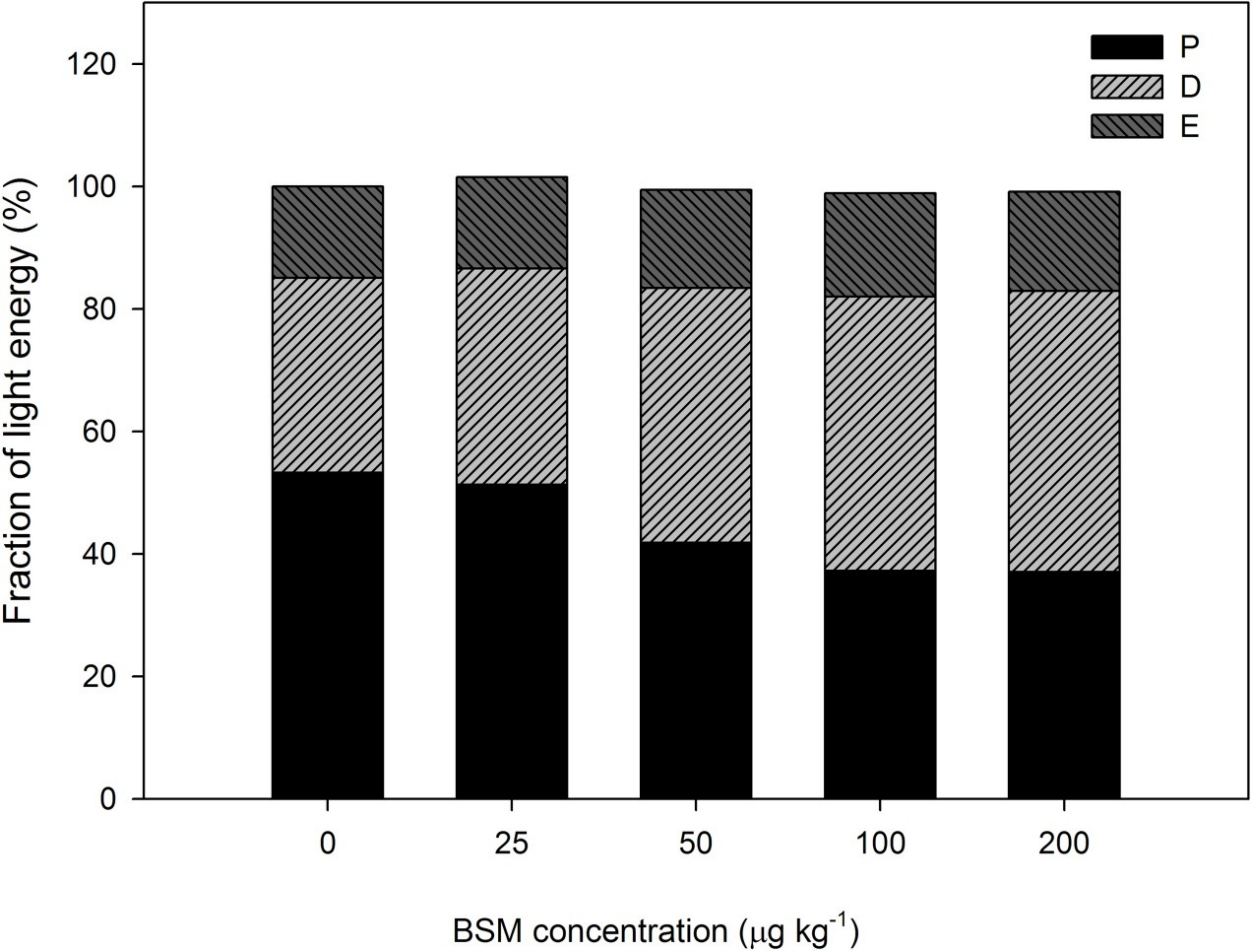
The fraction of photon energy absorbed by PSII antennae (P), the thermally dissipated portion (D), and the fraction of photonic energy absorbed by PSII antennae and trapped by “closed” PSII reaction centers (E) of cucumber seedlings under different concentrations (0, 25, 50, 100, and 200 μg kg^-1^) of the herbicide bensulfuron-methyl (BSM) in soil.

The energy partitioning pattern of absorbed light to the various pathways indicated that fraction P was decreased by the BSM residue, and fractions D and E increased in cucumber seedlings. Compared to the control, P decreased by 4–30% at different BSM concentrations, but D and E increased by 11–44% and 0–13%, respectively (Fig 5).

## Discussion

The shoot length, root length, SDM, RDM, and TDM of cucumber seedlings decreased significantly with the presence of the BSM residue, and the degree of decrease was directly related to the BSM concentration added to soil. The R:S ratio in seedlings initially decreased and then increased with rising BSM concentrations. The BSM residue had a more pronounced effect on RDM than SDM at lower concentrations. As the BSM residue concentration increased, the growth of both above- and belowground parts of cucumber seedlings were severely suppressed. These results were consistent with the results of Su *et al*.[9], but inconsistent with those of Su *et al*. [10]. Su *et al*.[10] reported that the inhibition of the root length of soybean and peanut were greater than those of plant height in the presence of BSM residue. This indicated that different crops responded differently to BSM residues. The reasean may be that at the lower concentrations, the critical threshold that the cucumber seedlings can tolerate has not yet been reached, so the BSM residue had a more pronounced effect on RDM than SDM. With the increase of the BSM residue concentration, it exceeds the critical threshold that the cucumber seedlings can tolerate, so the root and stem were significantly inhibited.

Abiotic stress factors such as high or low temperatures, strong or weak light intensity, and heavy metals are known to affect plant photosynthesis[15,24–25]. Photosynthetic pigments in plants absorb light energy and using it for photosynthesis. Chlorophyll content is directly related to the light energy conversion of photosynthesis. Some previous studies have suggested that pesticides significantly reduced the content of photosynthetic pigments and *P*_N_ [9,26]. In order to confirm the changes of photosynthesis caused by the BSM residue, we analyzed the *P*_N_ and other gas exchange parameters of cucumber seedlings under different concentration treatments. This induced a strong inhibition of net photosynthesis rate while reducing stomatal conductance, transpiration, and SPAD values. In contrast, *R*_D_ increased under the same conditions in cucumber seedlings. The leaf SPAD value was positively correlated with chlorophyll content[27]. These results showed that BSM treatment led to decreases in *P*_N_ and chlorophyll content, and the decline in chlorophyll content may also be one major reason for the reduction in *P*_N_. The reduction in *P*_N_ and the rise in *R*_D_ in cucumber seedlings suggested that enhanced respiration may be a mechanism to reduce the damage to photosynthesis caused by BSM residue[28–30].

Our results clearly showed that BSM treatment caused significant decreases in F_v_/F_m_, Φ_PSII_, qP, and ETR in cucumber seedlings, but an increase in F_o_. F_v_/F_m_ represents the primary light energy conversion efficiency of PSII. Previous studies have suggested that F_v_/F_m_ is a sensitive indicator of photosynthesis in plants under stress, a measure of the maximum capacity of primary light capture, reflecting the potential maximum photosynthesis. Reduction of F_v_/F_m_ indicated damage to an important portion of the PSII reaction center[31]. Φ_PSII_ and ETR decreased under BSM treatment mainly because BSM decreased the efficiency of excitation energy capture of open PSII reaction centers[32]. The decrease in qP suggested that BSM harmed PSII reaction centers and promoted the proportion of closed PSII reaction centers, probably generating a decrease in the proportion of available excitation energy used for photochemistry[33]. Previous studies have demonstrated that F_o_ is one of the criteria for estimating the number of antenna pigments of the PSII reaction center. The rise of F_o_ suggested that BSM led to damage to PSII reaction centers. In general, changes in F_v_/F_m_ and F_o_ can be used to diagnose whether photoinhibition occurs[34]. Our results agreed with the finding of Zhang *et al*.[35] that fenoxaprop-P-ethyl caused a significant inhibition of PSII in *Perilla frutescens*.

With respect to the allocation of excitation energy absorbed in PSII antennae, there are three pathways for its consumption. First, D represents the fraction of excitation energy dissipated in the PSII antennae, and was much larger in BSM-treated than control plants (Fig 5). Second, P represents the fraction of excitation energy utilized for photosynthetic electron transport, and was much lower in BSM-treated than control plants, consistent with impaired electron transport in BSM-treated plants. Third, E represents the fraction of excitation energy neither dissipated in PSII antennae nor employed for photosynthetic electron transport, and this slightly rose in BSM-treated plants. The increase in E may lead to de-excitation of chlorophyll in the least desirable path, potentially resulting in damaging singlet oxygen formation, which could damage thylakoid membrane components. The damage to PSII reaction centers in BSM-treated plants was indicated by the decrease in F_v_/F_m_ (Fig 4A).

Cucumber seedlings were negatively affected by the BSM residue in soil. When the residue of bensulfuron-methyl in soil exceeded 50 μg kg^-1^, the growth and photosynthetic capacity of cucumber was significantly inhibited. The BSM residue caused decreased photosynthesis, severely reduced plant height, root length, SDM, RDM, and TDM. Rice or wheat fields sprayed with BSM are not suitable for planting cucumbers in succession or rotation. Our results will provide the theoretical guidance for cucumber production.

## Supporting information

S1 Data This is the raw data. (XLSX)
